# The interplay between the pyramidalization of carbonyl carbons and the n → π* interactions in biomolecules

**DOI:** 10.1002/pro.70602

**Published:** 2026-05-04

**Authors:** Luigi Vitagliano, Luciana Esposito

**Affiliations:** ^1^ Institute of Biostructures and Bioimaging, CNR Naples Italy

**Keywords:** carbonyl carbon pyramidalization, conformational pre‐organization, conformation‐dependent geometry, non‐covalent interactions in biomolecules

## Abstract

The structural preferences of biomolecules arise from a delicate balance of different forces whose identification and quantification are challenging. Studies conducted over the last two decades have shown that the n → π* interaction formed between consecutive carbonyl groups plays a crucial role in stabilizing biomolecules, which exhibit a wide range of chemical properties and structural complexities. These studies also demonstrate that the n → π* interaction relies heavily on the pyramidalization of the carbonyl group, which accepts electrons, to such an extent that pyramidalization has frequently been considered a signature of this interaction. Here, statistical surveys of different structural databases aimed at evaluating the precise relationship between the n → π* interaction and the carbonyl carbon pyramidalization (*θ*_C_) show that the latter occurs independently of the presence of the former. Nevertheless, the n → π* interaction influences the degree of pyramidalization and affects its geometry. Importantly, our data indicate that the n → π* interaction occurs only when the local conformation of the accepting carbonyl group predisposes it for the required pyramidalization. The interplay between the n → π* interaction and *θ*_C_ pyramidalization underscores the importance of conformational pre‐organization in promoting specific interactions in biomolecules.

## 1 INTRODUCTION

Biomolecules generally operate within the intricate environment of living organisms by adopting specific structural states. Their ability to form particular three‐dimensional shapes is essential for interacting with ions and other biomolecules (Bartlett et al., 2013). Depending on their size, energetically favorable states are achieved through local or long‐range interactions. Despite decades of research, identifying and quantifying the role of non‐covalent interactions in shaping or influencing the three‐dimensional structure of these molecules remains challenging, even for simpler biomolecules (Dill & MacCallum, 2012). Recently, it has been suggested that, beyond well‐known forces such as hydrogen bonds and electrostatic or van der Waals interactions, biomolecular structures are stabilized by minor yet essential effects known as secondary forces (Jena et al., 2022; Newberry & Raines, 2019). Among these, the interaction between carbonyl groups and nucleophiles (including other carbonyl groups) is particularly notable, involving contacts where the nucleophile donates a lone pair (n) electron density into the empty π* orbital of the carbonyl (n → π* interaction) (Newberry & Raines, 2017). Over the past two decades, research has demonstrated that this interaction plays a crucial role in determining the structural preferences of many organic compounds containing carbonyl or thiocarbonyl groups, as well as of macromolecules such as polypeptides and proteins (Bartlett et al., 2010; Choudhary & Raines, 2011a; Choudhary & Raines, 2011b; DeRider et al., 2002; Dones et al., 2021; Fiala et al., 2023; Fufezan, 2010; Gorske et al., 2007; Hanna et al., 2025; Hinderaker & Raines, 2003; Hodges & Raines, 2006; Kilgore et al., 2020; Newberry et al., 2013; Newberry et al., 2014; Newberry & Raines, 2014; Newberry & Raines, 2017; Singh et al., 2016; Singh & Das, 2015; Wilhelm et al., 2014; Windsor et al., 2019). These studies have also distinguished the contribution of this donation from purely electrostatic interactions, such as dipole–dipole forces (Kamer et al., 2013). One observable effect of the n → π* interaction is the pyramidalization of the carbonyl carbon (*θ*_C_), which facilitates the interaction. It has been proposed that the displacement of the acceptor carbonyl carbon toward the donor and away from the plane formed by the three covalently linked atoms is a characteristic feature of this interaction, serving as a signature of the n → π* interaction (Choudhary & Raines, 2011b; Newberry et al., 2013; Newberry et al., 2014; Panwaria & Das, 2022; Singh & Das, 2015).

Along with other possible distortions of the amide bond—such as deviation of the ω angle from planarity (Edison, 2001) and nitrogen pyramidalization—the pyramidalization of the carbonyl carbon is one of the well‐studied modes of amide bond distortion in proteins and peptides (Berkholz et al., 2009; Berkholz et al., 2012; Esposito et al., 2000; Esposito et al., 2005). High‐resolution crystallographic structures of peptides and proteins have revealed that many small but significant deviations from Pauling's paradigm on peptide bond planarity, a fundamental concept in structural biology, exist (Improta et al., 2011). These findings suggest that the distortion from planarity is heavily dependent on the local conformation of the protein or peptide (Balasco et al., 2017). Notably, an evident 120° periodicity in these distortions was observed based on the main chain ψ dihedral angle. This periodicity was initially identified in small molecules (Cieplak, 1985; Jeffrey et al., 1985). Using ultrahigh‐resolution structures, we demonstrated its presence in proteins (Esposito et al., 2000; Esposito et al., 2005), and subsequently observed it in special contexts such as peptides forming amyloid‐like fibers (Esposito et al., 2013). Similar trends were later reported by other groups (Brunner et al., 2021; Brunner et al., 2025).

This conformation‐dependent *θ*_C_ pyramidalization has also been studied using quantum chemistry on various models, including an Ala residue in a peptide‐like system, in different solvation environments (Improta et al., 2011). These computational results closely match the statistical surveys, showing a similar dependence of *θ*_C_ pyramidalization on the ψ dihedral angle. Notably, in peptide systems, the ψ angle modulates both N n → CO π* and C^α^—X σ → CO π* interactions, where the X indicates any single‐bonded substituent of the C^α^ atom (other than the carbonyl group). In peptides, due to the presence of the substituents on the C^α^ atoms, the maximization of the N n → CO π* interaction, which is responsible for the Pauling resonance, does not always coincide with the perfect planarity of the peptide bond. Indeed, for specific states for which a significant pyramidalization was observed in the experimental structures (see, for instance, the ψ = 150° conformation), the optimization of these orbital interactions occurs upon a small but significant distortion of the peptide bond. Also, the maximization of the C^α^—X σ → CO π* interaction energy is a driving factor for the distortion. Indeed, for each specific C^α^—X bond, the σ → π* interactions are maximized when the X substituent is perpendicular to the amide plane (ψ = ±150°, ±90°, ±30°) (Improta et al., 2011).

The overall effect is that the carbonyl C atom adopts a partial sp^3^ character through a pyramidalization toward staggered geometries around the C^α^—C bond, with the pyramid's apex at the carbonyl C atom being anti to the C^α^—X bond nearly perpendicular to the peptide plane. This results in a clear sinusoidal dependence on ψ, with *θ*_C_ alternating between positive and negative values every 60° of the dihedral angle.

In this scenario, depending on the perspective of literature studies, the origin of the *θ*_C_ pyramidalization could have a dual cause, as it might be induced either by non‐bonded interactions (n → π* donation) or by local conformational effects. To gain insights into this puzzling issue, we investigated the impact of local conformation and n → π* donation on *θ*_C_ pyramidalization across various biomolecular systems with different complexities and chemical properties.

## 2 RESULTS AND DISCUSSION

The well‐established observation that, in proteins and peptides, the *θ*_C_ pyramidalization is dictated by the local conformation prompted us to investigate whether similar effects could be observed in other carboxylic acid derivatives in which an electronegative atom is directly attached to the carbon of the carbonyl group.

**FIGURE 1:**
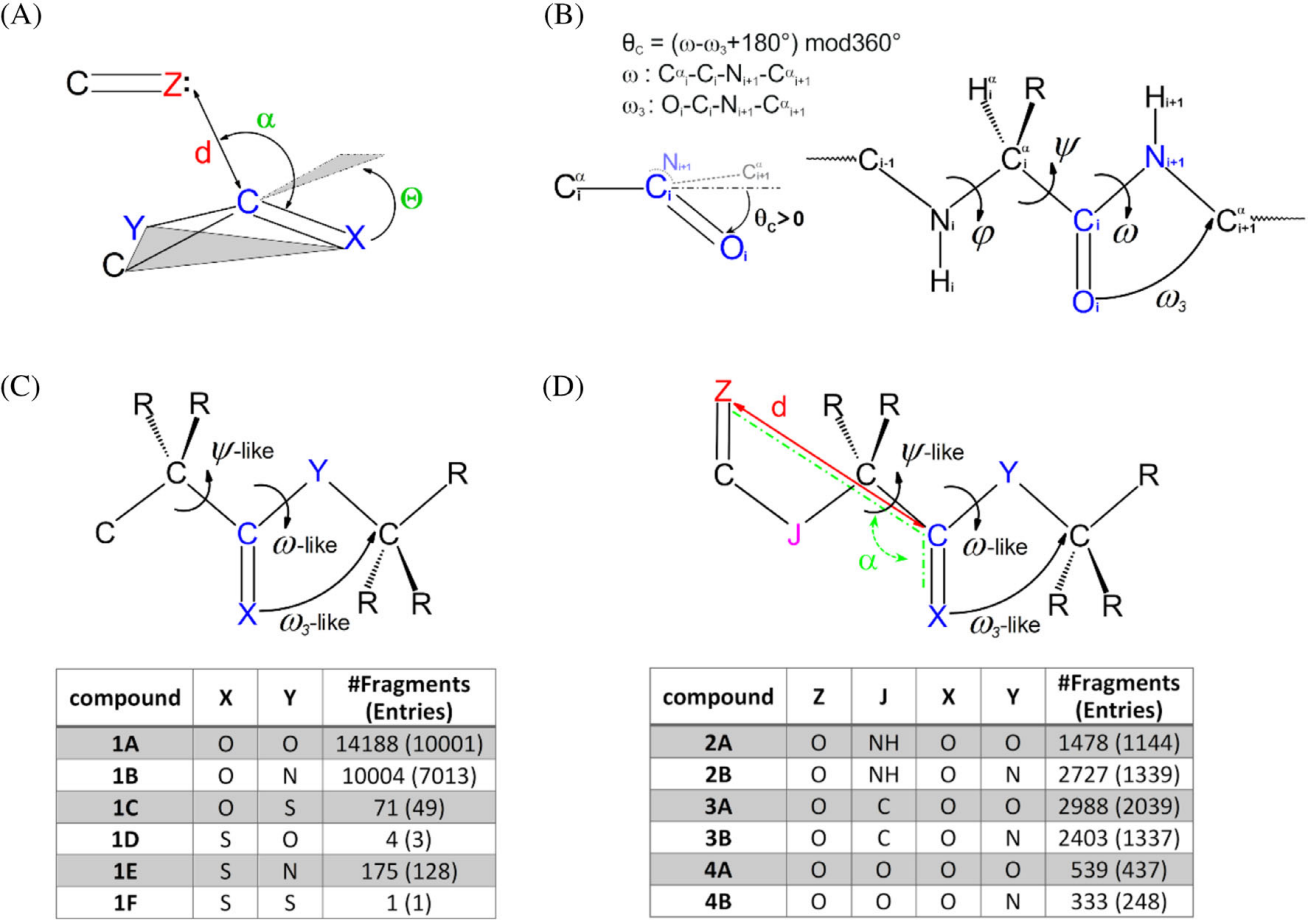
Schematic representations of C pyramidalization and searched fragments. (a) Geometrical parameters used by Raines and coworkers to characterize C pyramidalization (*Θ*) and n → π* interactions (*d*, *α*). The sign of *Θ* is considered to be positive when the carbonyl C pyramidalizes toward the donor Z, just like in figure. (b) Carbonyl C pyramidalization definition (*θ*_C_) as measured by differences of dihedral angles in proteins. In the lower left, the view is along the C—N_i+1_ bond and the positive sign of *θ*_C_ parameter is shown. (c) Searched compound 1A‐1F with specified number of structures and fragments analyzed. (d) Searched compound 2A‐4B with specified number of structures and fragments analyzed in order to examine the effect of n → π* interactions displayed by vicinal C—Z groups. The three dihedral angles are illustrated: J—C—C—Y ψ‐like, C—C—Y—C ω‐like, X—C—Y—C ω_3_‐like.

Among the two distinct operative definitions of the carbonyl carbon pyramidalization reported in the literature (Figure 1a,b) (Burgi et al., 1973; Choudhary et al., 2009; Cieplak, 1985; Winkler & Dunitz, 1971), we used the one which measures the *θ*_C_ pyramidalization as the difference between two dihedral angles (ω and ω_3_) as illustrated in Figure 1b (for details see Section 4.1).

As detailed in Table S1, these analyses were conducted using structural databases of small organic molecules (Cambridge Structural Database, CSD) and protein structures (Protein Data Bank, PDB). As illustrated in the following subsections, we adopted a stepwise approach, beginning by revisiting the conformation‐dependent *θ*_C_ pyramidalization in the carboxylic acid derivative. We then evaluated the impact of the n → π* donation on the *θ*_C_ pyramidalization. Finally, based on the findings from these analyses, we (re)interpreted the occurrence of this pyramidalization in some particularly interesting systems (mixed α/β peptides, polyproline/collagen triple motifs, and globular proteins).

### 2.1 The conformation‐dependent *θ*_C_ pyramidalization among carboxylic acid derivatives

Initial surveys of the CSD were conducted to identify carboxylic acid derivatives within carbon‐rich environments (Figure 1c), aiming to reduce local interactions between these functional groups and other electronegative atoms. Depending on the chemical nature of the X and Y atoms, the database was searched for esters (1A), amides (1B), thioesters (1C),  thionoesters (1D), thioamides (1E), and dithioesters (1F).

As shown in the inset of Figure 1c, a large number of fragments belonging to the ester (1A—14,188 fragments from 10,001 structures) and amide (1B—10,004 occurrences from 7013 structures) groups were identified. Conversely, compounds containing thioesters (1C) are quite rare, with only 71 identified across 49 structures. Replacing the C=O bond with the C=S bond significantly decreases the number of detected fragments. In this group of compounds, a significant number of fragments were detected only for the thioamide group (1E) (175 out of 128 entries), whereas thionoesters (1D) and dithioesters (1F) were nearly absent from the database.

**FIGURE 2:**
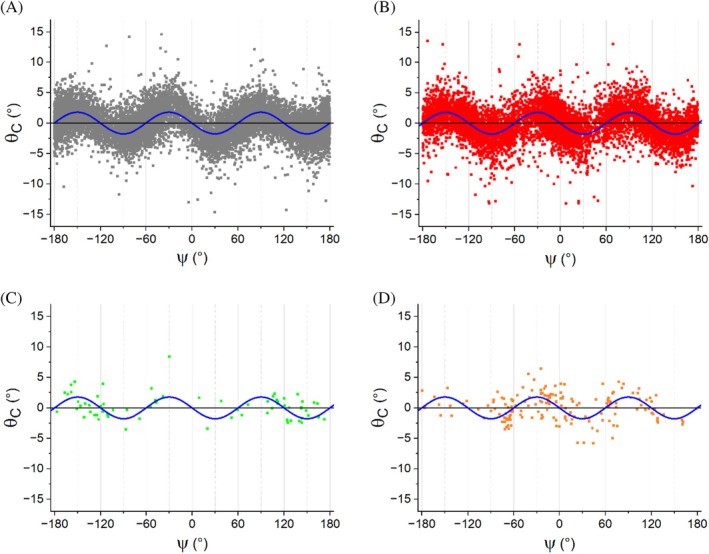
Carbon pyramidalization *θ*_C_ angle versus ψ‐like (ψ) dihedral angle in (a) Ester groups of 1A compounds (X, Y=O). (b) Amide groups of 1B compounds (X = O, Y = N). (c) Thioester groups of 1C compounds (X = O, Y = S). (d) Thioamide groups of 1E compounds (X = S, Y = N). Data in panel A were fitted by a sine function, resulting in an amplitude of 1.8° (blue line). This line has been superimposed on the data in all the other panels.

Based on these observations, we examined the values of *θ*_C_ pyramidalization as a function of local conformation for the significantly populated fragments (esters, amides, thioesters, and thioamides). Specifically, we plotted the *θ*_C_ pyramidalization against the dihedral angle CCCY, which we referred to as a ψ‐like angle because it resembles the ψ angle found in proteins and peptides (Figure 1b). As shown in Figure 2a,b, the pyramidalization value shows a strong and regular dependence on the ψ‐like angle, with a periodicity of 120° for both amides and esters. Due to this pattern, *θ*_C_ exhibits positive and negative values, alternating signs every 60° of ψ‐like. It is important to note that within this trend, a significant variation of *θ*_C_ observed values occurs for a given ψ‐like value. This spread of *θ*_C_ values indicates that the local environment influences the degree of pyramidalization. However, the dependence of pyramidalization on local conformation remains evident, despite the significant chemical diversity of the compounds in each dataset.

Although, as mentioned above, the limited data available for the subclass of compounds containing the C=S bond do not allow for a detailed analysis, the available data for the thioamide group suggest similar trends. Indeed, in regions characterized by ψ‐like values falling in the range from −60° to 150°, the observed extent of pyramidalization is consistent with that seen in esters and amides. In this interval, the average value of *θ*_C_ observed for the thioamide shows the typical alternation of signs. Negative values are observed for the ψ‐like intervals 0°–60° (average *θ*_C_ = −0.74 on 33 points) and 120°–180° (average *θ*_C_ = −1.02 on 13 points), while positive values are seen in the intervals −60° to 0° (1.21 on 47 points) and 60°–120° (0.78 on 33 points). No clear trends emerge in other regions where the collected data are limited.

Globally, these findings demonstrate that the dependence of carbonyl carbon pyramidalization on local conformation, previously observed in peptides and proteins (Esposito et al., 2000; Esposito et al., 2005; Jeffrey et al., 1985), is a widespread phenomenon occurring in various compounds containing carbonyl groups. As suggested for amides (Esposito et al., 2000; Windsor et al., 2019), it is also plausible that the conformation‐dependent pyramidalization may influence the reactivity of esters. Based on the data presented here, it can be inferred that in reactions involving this functional group that proceed through sp^3^ states, the proper alignment of the ψ‐like angle may facilitate the formation of the transition state.

### 2.2 The delicate interplay between the *θ*_C_ pyramidalization and the n → π* interaction

To evaluate the impact on pyramidalization caused by an extra nearby carbonyl group that could act as a donor in n → π* interactions, we surveyed the CSD structures by looking for fragments containing two distinct carbonyl groups. As shown in Figure 1d, we explored various combinations of the O=C—C, ester, and amide groups (Table S1). Our survey shows that the most common combinations in the CSD involve an O=C—N group, followed by either an ester (2A) or another amide (2B), or an O=C—C group preceding an ester (3A) or an amide (3B). Significantly populated are the O=C—O/ester (4A) and O=C—O/amide (4B) combinations. Finally, over 30 occurrences are observed for the O=C—N/thioamide (5B—cases 31), S=C—N/ester (8A—cases 36), and S=C—N/amide (8B—cases 69) combinations. These fragments were dissected based on the occurrence of the n → π* interaction. As detailed in Section 4.2, the occurrence of the donation n → π* was assessed based on the geometric operational definition used by Raines and collaborators (Bartlett et al., 2010) (Figure 1a and Table S1). The interaction was considered to be present if the Z…C=X angle (α) falls in the interval 99°–119° and the Z…C distance was lower than or equal to 3.22 Å or 3.45 Å for Z=O or Z=S, respectively.

**FIGURE 3:**
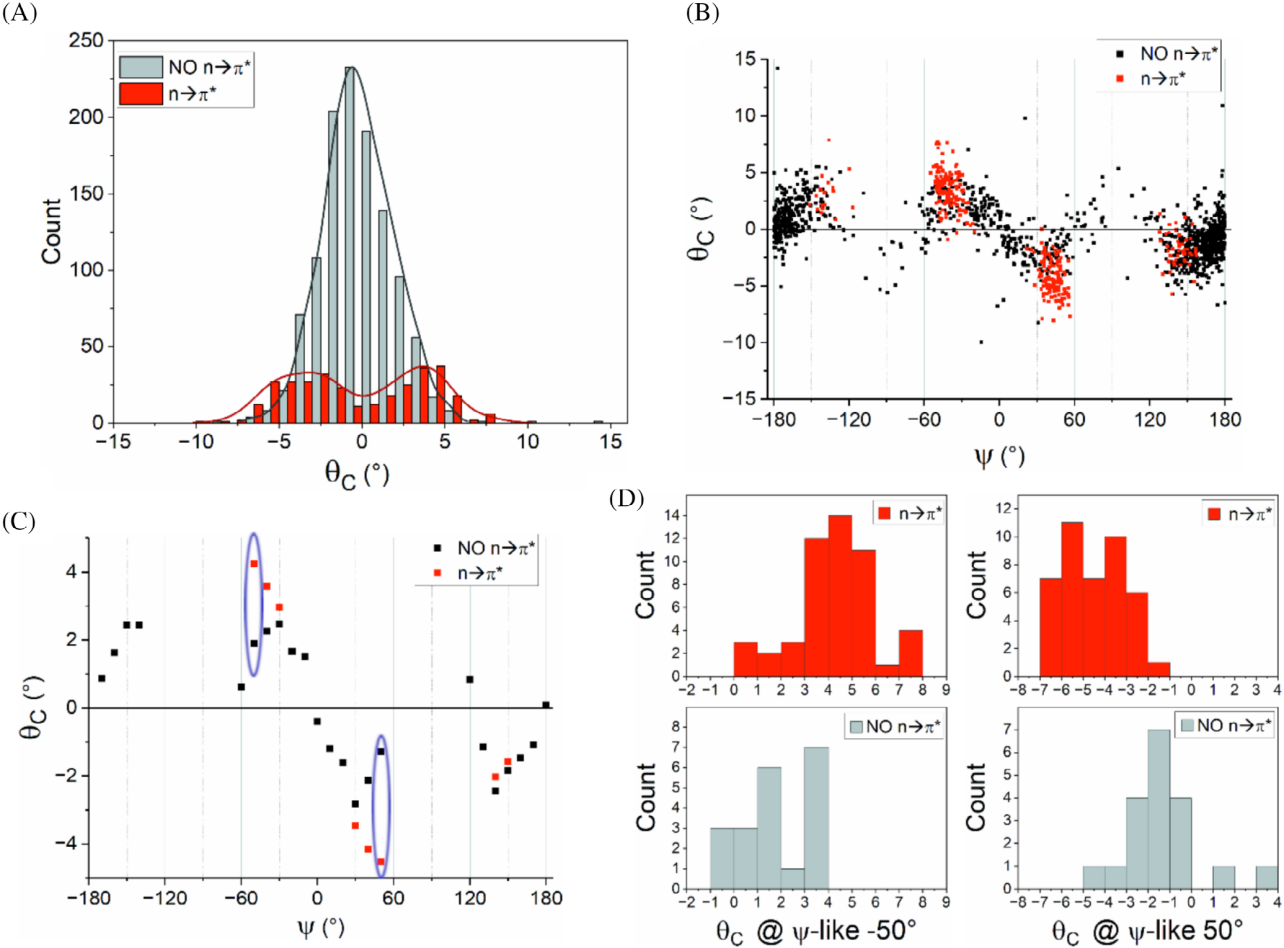
Carbon pyramidalization *θ*_C_ angle versus ψ‐like (ψ) dihedral angle in 2A compounds. (a) Distributions of *θ*_C_ values for the two classes of carbonyls (in red are the carbonyls which have a nearby carbonyl group establishing n → π* interactions, in black those which have not, NO n → π*). A fitting curve (kernel smoothing) is superimposed to the histograms. (b) Plot of *θ*_C_ vs. ψ‐like for all the fragments selected. (c) Averaged pyramidalization per bin (10°) for points in panel B (only bins containing more than 10 measures are considered). Selected different values for the two classes in the same bin are circled in blue and analyzed in panel D. (d) *θ*_C_ distributions for the ψ = −50° and ψ = 50° bins of panel C for both carbonyls displaying n → π* interactions (red‐upper panel) and carbonyls not showing them (gray‐lower panel). See Tables S1‐S3 for details on the populations of the overall datasets (Table S1), of the plotted bins (Table S2), as well as on the statistical significance of the mean differences (Table S3).

The analysis of the distribution of *θ*_C_ pyramidalization values for fragments containing the amide/ester combination (2A), examined as a function of the occurrence of the n → π* interaction, reveals notable differences. Indeed, while the fragments where this interaction is not present show a Gaussian‐like distribution centered around 0° (Figure 3a), the presence of the n → π* interaction results in a bimodal distribution (Figure 1a) with a minimum for values close to *θ*_C_ = 0°. The trend of *θ*_C_ pyramidalization as a function of the ψ‐like parameter for these fragments, shown in Figure 3b,c, exhibits the expected pattern with a 120° periodicity, similar to that observed for fragments 1A (Figure 2a). Although periodicity appears regardless of the presence of the n → π* interaction, visual analysis of these plots indicates that, in fragments where donation occurs, the absolute value of *θ*_C_ tends to be higher. Additionally, the ψ‐like value at which pyramidalization occurs is shifted, as shown in Figure 3c where the averaged pyramidalization per ψ‐like bin (10°) is reported for the sufficiently populated bins (see Table S2). To evaluate the significance of these observations, we compared the distribution of the *θ*_C_ values for fragments with or without the donation in ψ‐like ±50° bins where large differences are found (Figure 3d). The comparison of these distributions indicates that in fragments with the n → π* interaction, larger pyramidalizations are observed. The significance of the differences between the two distributions has been statistically evaluated (Table S3). As shown in Table S3, the probability that two distributions generated with random values exhibit the observed mean differences for the two distributions in Figure 3d is as low as 2.7 × 10^−9^ for ψ‐like = 50° and 4.4 × 10^−7^ for ψ‐like = −50°.

**FIGURE 4:**
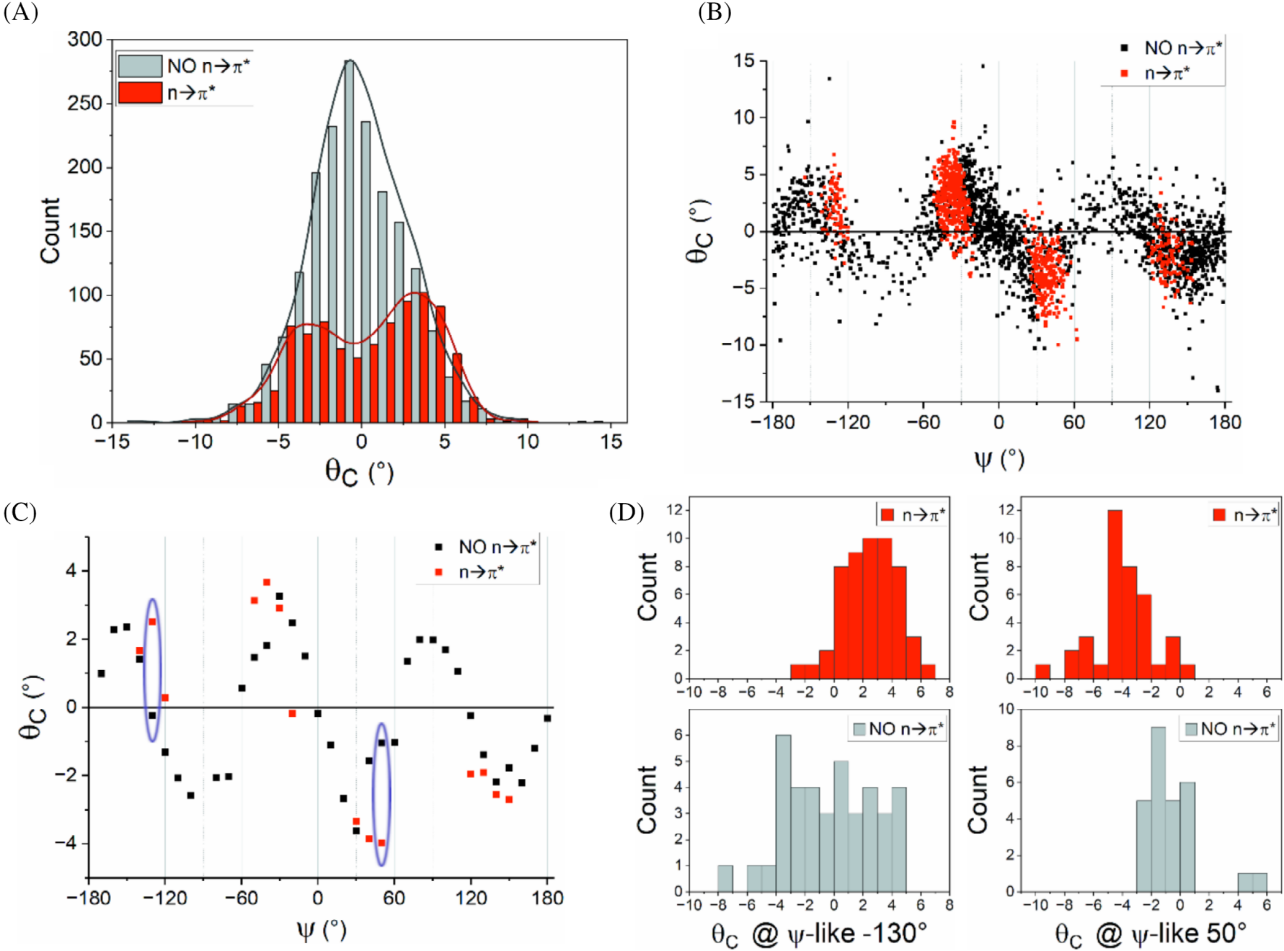
Carbon pyramidalization *θ*_C_ angle versus ψ‐like (ψ) dihedral angle in 2B compounds. (a) Distributions of *θ*_C_ values for the two classes of carbonyls (in red are the carbonyls which have a nearby carbonyl group establishing n → π* interactions, in black those which have not, NO n → π*). A fitting curve (kernel smoothing) is superimposed on the histograms. (b) Plot of *θ*_C_ versus ψ‐like for all the fragments selected. (c) Averaged pyramidalization per bin (10°) for points in panel B (only bins containing more than 10 measures are considered). Selected different values for the two classes in the same bin are circled in blue and analyzed in panel D. (d) *θ*_C_ distributions for the *ψ* = −50° and *ψ* = 50° bins of panel C for both carbonyls displaying n → π* interactions (red‐upper panel) and carbonyls not showing them (gray‐lower panel). See Tables S1‐S3 for details on the populations of the overall datasets (Table S1), of the plotted bins (Table S2), as well as on the statistical significance of the mean differences (Table S3).

The trends observed for compounds of class 2B (amide/amide) (Figure 4) closely resemble those observed for compounds 2A. Once again, the presence of the n → π* interaction is linked to changes in the overall distribution of the *θ*_C_ values (Figure 4a), a shift in the peaks and minima in the ψ‐like plot (Figure 4b,c), and higher values at the extremes (Figure 4c,d and Table S3). Similar tendencies are observed for compounds where the O=C—C group is followed by ester (compounds 3A) and amide (3B) groups (Figures S1 and S2 and Table S3), although in these fragments, especially for the O=C—C/amide combinations, the number of occurrences where the n → π* interaction is present is relatively small (Table S2).

Although minimal data are available for the ester/ester (compounds 4A) and ester/amide (4B) combinations (Table S1), which preclude statistical analyses, inspection of the plots in Figure S3 suggests similar trends.

Collectively, the data reported here convincingly indicate that although the *θ*_C_ pyramidalization may occur independently of the n → π* donation, this interaction significantly affects the peptide bond deformation.

We further investigated whether, in molecules with more than two consecutive carbonyl groups, cooperative effects could enhance the pyramidalization. In particular, we verified whether a donor carbonyl group in peptide‐like systems can induce greater pyramidalization when it also serves as an acceptor in an n → π* interaction. As shown in Figure S4, the involvement of the carbonyl group in multiple n → π* interactions generally results in greater pyramidalization. Although the observed effects are limited and further investigations are required, this observation suggests that cooperative effects operate in biomolecules containing several consecutive carbonyl groups that are geometrically oriented for donation, as in polyproline stretches present in proteins (see also below).

### 2.3 (Re)interpretation of observed pyramidalization in some special cases

The interplay between local conformation, *θ*_C_ pyramidalization, and n → π* interaction was also assessed in the following paragraphs for biomolecular systems for which this subject was previously investigated.

#### 2.3.1 *θ*_C_ pyramidalization in mixed α/β peptides

**FIGURE 5:**
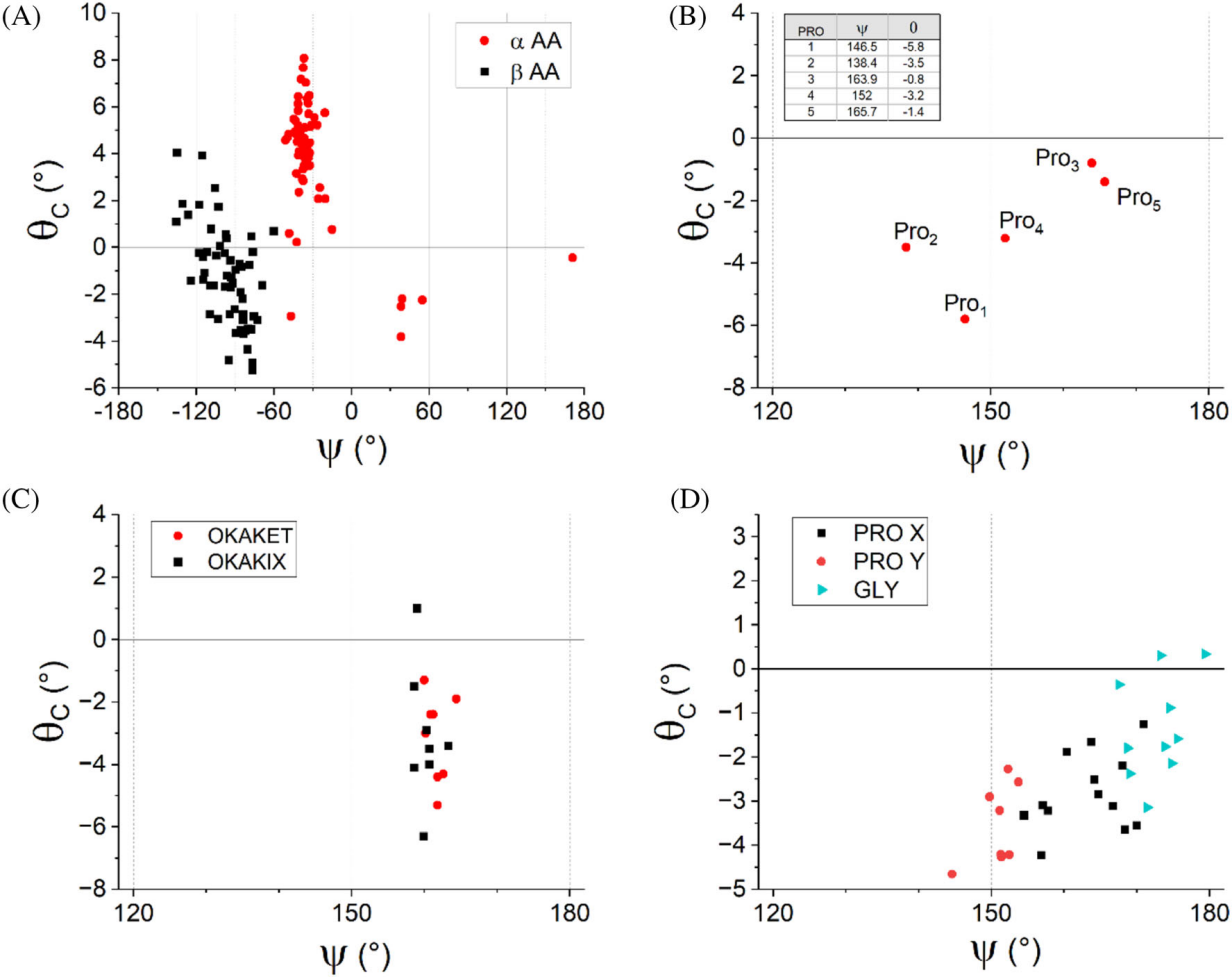
Special cases of conformation‐induced carbonyl carbon pyramidalization. (a) Pyramidalization in peptides adopting a helical structure and containing both α‐ and β‐amino acid residues (in red and black, respectively). The analyzed 19 peptide structures are extracted from the paper by Choudhary and Raines (Choudhary & Raines, 2011b). (b) Pyramidalization in an oligoproline PPII‐Helix (CCDC entry SOWJUL) by Wennemers and coauthors (Wilhelm et al., 2014). (c) Pyramidalization in two hexaproline model peptides representing a ligand in metal–organic frameworks (Schnitzer et al., 2021). The structures (CCDC entries OKAKET and OKAKIX) contain two hexaproline molecules in the asymmetric unit. We excluded from the analysis the disordered Pro residues. We also excluded Pro residues adjacent to the aromatic carboxylate at the N‐terminus that coordinates to the metal ions thus possibly inducing a considerable strain; (d) Pyramidalization in a collagen model peptide in triple helix determined at high resolution (PDBcode 6SYJ) (Maaßen et al., 2020). Only selected residues have been considered by excluding non‐canonical, disordered, and terminal amino acids.

A strong correlation between *θ*_C_ pyramidalization and the n → π* interaction was proposed based on an analysis of 19 peptide structures containing both α‐ and β‐amino acid residues, assuming a common α‐helical structural motif (Choudhary & Raines, 2011b). In particular, it was shown that α‐amino acid residues exhibit dramatic pyramidalization, whereas β‐amino acid residues do not. This observation was correlated with the observation that n → π* interaction can occur in the α‐helix formed by α‐amino acid residues. Still, the O and the C atoms that should be involved in the donation are too far apart in the structures formed by β‐amino acid residues. We revisited the topic by evaluating the *θ*_C_ pyramidalization of residues in these 19 peptides, listed in Table S4, as a function of the local conformation, specifically the ψ angle. As shown in Figure 5a, α‐ and β‐amino acid residues exhibit quite different ψ angles in these helical motifs. The inspection of the figure demonstrates that different local conformations of the residues are responsible for the sign and the extent of the *θ*_C_ pyramidalization in these mixed peptides. The distribution of points in the plot, which fully conforms to the ψ‐like dependency described earlier, shows that the high pyramidalization of α‐ compared to β‐amino acid residues results from the clustering of the former around ψ values (~−40°), corresponding to high positive pyramidalizations. In contrast, the β‐amino acid residues occupy regions in the ψ space with both positive and negative values, although significant deformations of the peptide bonds are also observed for these residues.

#### 2.3.2 *θ*_C_ pyramidalization in high‐resolution polyproline II and collagen structures

The polyproline (PPII) motif is a widespread feature of proteins, both in their folded and denatured states, and, unlike other secondary structure elements, is not stabilized by hydrogen bonding. Although the PPII motif has been identified in several protein structures, its atomic‐level characterization remained elusive (Williamson, 1994) until a few years ago (Wilhelm et al., 2014). This highly accurate PPII structure, containing the oligoproline peptide (Pro)_6_, allows us to further examine the conformation–pyramidalization paradigm. As reported in Figure 5b, the five Pro residues for which it is possible to define the ψ angle have values for this dihedral angle that fall in the range of approximately 138°–166°. These residues exhibit negative *θ*_C_ pyramidalization values, consistent with the conformation–pyramidalization paradigm. Moreover, within this framework, the largest values are observed for residues such as Pro1, Pro2, and Pro4, whose ψ angles are close to ~150°, as those associated with high pyramidalizations in the amide model compounds (Figure 2b).

More recently, Wennemers and coworkers have reported additional structures of this motif where the hexaproline serves as a ligand in metal–organic frameworks (MOF) (Schnitzer et al., 2021). As shown in Figure 5c, for these structures, the points are concentrated within a narrow range of ψ values; therefore, no dependence of pyramidalization on the conformation could be studied, although the observed values align with the *θ*_C_ value and sign at the specific ψ angle (~160°).

The correlation between *θ*_C_ pyramidalization was further examined in an ultrahigh‐resolution collagen structure because, in the triple helix, the individual chains adopt a PPII conformation but are held together by a well‐defined pattern of hydrogen bonds. Interestingly, recent studies, conducted by both Raines and Wennemers groups, have shown the significance of the n → π* interaction in stabilizing the triple helix structure (Schnitzer et al., 2025; Wilhelm et al., 2014; Yang et al., 2024). The impact of the local ψ angle on the *θ*_C_ pyramidalization was evaluated here using the structure of a collagen‐like peptide determined at 0.81 Å resolution (Protein Data Bank entry 6SYJ) (Maaßen et al., 2020). As shown in Figure 5d, a strong correlation between the pyramidalization and the dihedral angle is evident, despite the chemical diversity of the amino acid residues involved. Therefore, despite the increasing complexity of collagen triple‐helix motifs compared to the individual PPII chain, the conformation‐pyramidalization paradigm is fully confirmed.

#### 2.3.3 *θ*_C_ pyramidalization in atomic resolution protein structures

**FIGURE 6:**
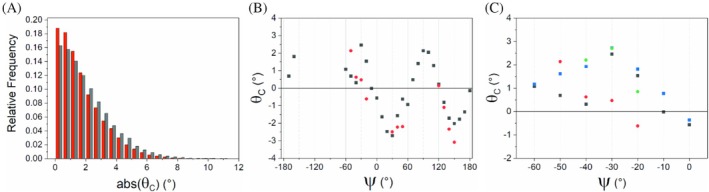
Pyramidalization in proteins. (a) Distributions of the absolute values of *θ*_C_ pyramidalization of residues in proteins. In red are shown the values for peptide groups which receive an n → π* interaction (*N* = 12,167), whereas in gray are those for groups that do not (*N* = 24,367). (b) Average values for *θ*_C_ pyramidalization over 10° bins of *ψ* values. Only values for bins with a population over than *N* = 40 are shown in the plot (see Table S5 for population details); peptides with n → π* present (red circle), peptides with n → π* absent (black square). (c) Close‐up view of *θ*_C_ as a function of *ψ* in the zone *ψ* = −60°–0° of panel B. We also separately plotted the values for peptides that are not in secondary structure (SS) elements (see Table S6 for bin population details). Peptides with n → π* present (red circle, all peptides; green circle, only peptides not in SS); Peptides with n → π* absent (black square, all peptides; blue square, only peptides not in SS).

The roles of the *θ*_C_ pyramidalization and the n → π* interaction have been examined in several previous studies (Bartlett et al., 2010; Choudhary et al., 2009; Newberry et al., 2014). Here, we investigated how conformation and the n → π* interaction affect pyramidalization using protein structures at atomic resolution (≤1.0 Å) (Figure 6). A simple analysis of the extent of pyramidalization in residues with and without the n → π* interaction shows that *θ*_C_ appears larger in residues where the donation is absent (Figure 6a). However, when *θ*_C_ pyramidalizations are reported as a function of the local ψ angle and the n → π* interaction, a quite different picture emerges. Indeed, as shown in Figure 6b, except for some bins in the ψ region within the −60° to 0° interval, the presence of the n → π* interaction generally increases pyramidalization. To interpret the anomalous data for the −60° to 0° interval, we analyzed it based on the structural features of the corresponding residues. The inspection of the resulting plot (Figure 3c) indicates that, when only peptides that are not embedded in a regular secondary structure are considered, the general expected trend is somehow restored. The unusual trend observed in this interval may be attributed to the flattening of the peptide bond, accompanied by a reduction in pyramidalization in protein α‐helical regions, which could also arise from the strong polarization of the peptide carbonyl groups in structural motifs (Lario & Vrielink, 2003). The specificity of α‐helical fragments, in which hydrogen bonding and the n → π* interaction simultaneously operate, may explain the lack of evidence observed by NMR for the donation (Worley et al., 2012). On the other hand, the enhanced pyramidalization observed for the ψ corresponding to PPII regions suggests that the n → π* interaction is important for stabilizing this element, as previously proposed (Horng & Raines, 2006; Wilhelm et al., 2014).

## 3 CONCLUSIONS

The implementation of predictive approaches based on machine‐learning techniques is revolutionizing structural chemistry and biology (Abramson et al., 2024; Gu et al., 2025; Jumper et al., 2021; Wang et al., 2025). Although they are remarkable tools for obtaining fundamental information to establish structure–function correlations, these approaches provide limited chemical insight into the forces that drive specific three‐dimensional arrangements of molecules. Recent investigations have highlighted the role that secondary forces, in addition to well‐known interactions such as hydrogen bonding and electrostatic/hydrophobic interactions, play in determining the structure and reactivity of biomolecules (Bartlett et al., 2010; Fufezan, 2010; Newberry & Raines, 2017; Newberry & Raines, 2019). A notable number of studies in the literature have emphasized the importance of the recently discovered n → π* interaction in shaping the structure of various types of biomolecules. These studies also demonstrate that the n → π* interaction heavily relies on the pyramidalization of the carbonyl group, which accepts electrons, to such an extent that pyramidalization has been frequently considered as a signature of this interaction. Since previous analyses have reported the importance of local conformation on the occurrence of the *θ*_C_ pyramidalization, we here surveyed different structural databases to assess the precise relationship between the n → π* interaction and this pyramidalization. Our data clearly indicate that, depending on the local conformation of the biomolecule in terms of the ψ‐like angle, a significant *θ*_C_ pyramidalization is detectable with a clear periodic trend which is the result of a coupled structural/electronic process (Improta et al., 2011). In this general background, the occurrence of n → π* interaction, which occurs only in specific structural states, significantly reinforces the conformationally induced pyramidalization. Notably, the direction of the extra pyramidalization produced by the n → π* interaction is the same as that induced by the local conformation. Therefore, the local conformation not only provides the geometric conditions for the n → π* donation but also predisposes the accepting carbonyl group to *θ*_C_ pyramidalization. The interplay between the n → π* interaction and *θ*_C_ pyramidalization highlights the importance of conformational pre‐organization in promoting specific interactions in biomolecules, demonstrating that a comprehensive characterization of the involved factors is crucial for understanding the mechanisms of action of biological molecules.

In conclusion, the observation that *θ*_C_ pyramidalization can be observed in the absence of the n → π* interaction suggests that it cannot be considered sensu stricto a signature of the phenomenon. A better definition of the signature of n → π* interactions is likely the fulfillment of the geometric criteria defined by Raines and coworkers in terms of nucleophile approaching distance (*d*) and angle (*α*) (Bartlett et al., 2010), accompanied by pronounced *θ*_C_ pyramidalization.

## 4 MATERIALS AND METHODS

### 4.1 Definition of the carbonyl carbon pyramidalization

Two distinct operative definitions of the carbonyl carbon pyramidalization are reported in the literature. The first is based on the displacement of carbonyl carbon out of the plane formed by the atoms to which it is covalently linked (Figure 1a) (Burgi et al., 1973; Choudhary et al., 2009). In this definition, the pyramidalization *Θ* angle is the angle between the carbonyl vector C=X and the plane containing C—C—Y atoms, as illustrated in Figure 1a. This *Θ* angle, measured by Raines & coworkers (Choudhary & Raines, 2011b), is considered to be positive when the carbonyl carbon pyramidalizes by its displacement toward the lone pair (n) donor (like the Z atom in Figure 1a), which establishes the n → π* interaction. The second definition, widely adopted for proteins and peptides, defines the *θ*_C_ pyramidalization as the difference between two dihedral angles (ω and ω_3_) as illustrated in Figure 1b (Cieplak, 1985; Winkler & Dunitz, 1971). The pyramidalization parameter is calculated as a difference of dihedral angles as follows: *θ*_C_ = (ω‐ω_3_ + 180°) mod360°. Applying this formula automatically gives to the angle *θ*_C_ a sign which indicates the apex direction of the pyramidal C carbon, and it is independent of the position of the possible nearby electron pair donor.

We use the *θ*_C_ angle to quantify C pyramidalization throughout our analysis. Additionally, we utilize the ψ‐like dihedral angle, which describes the rotation around the bond preceding the carbonyl group. It is denoted as the ψ‐like angle, in analogy with the notation used for describing protein backbone dihedral angles.

### 4.2 Analysis of the Cambridge structural database—selection of the fragments

The search for organic molecules containing derivatives of carboxylic acids (esters, thioesters, amides, and thioamides) present in the CSD (version 6.0, updated Apr 2025) was conducted using the CCDC ConQuest and Mercury software. A dataset of highly accurate CSD structures was generated, excluding crystallographic models with *R* > 5% and those containing either errors or crystallographic disorder. The Conquest software was also used to calculate the geometrical parameters (by the 3D option “Define Parameters”) for distances, angles, and torsions needed for the analysis.

Distinct surveys of the CSD were conducted to investigate different aspects of carbon carbonyl pyramidalization and its occurrence in various classes of compounds. Initial searches were performed on individual derivatives of carboxylic acid that were embedded in a chain of carbon atoms (Figure 1c). Mutatis mutandis at the positions X and Y of Figure 1c, the searches were performed to identify fragments corresponding to esters (X and Y = O—compounds 1A), amides (X = O and Y = N—compounds 1B), and thioesters (X = O and Y = S—compounds 1C). We then extended the analysis to similar molecules with the sulfur atom replacing the carbonyl oxygen, that is, to thionoesters (X = S and Y = O—compounds 1D), thioamides (X = S and Y = N—compounds 1E), and dithioesters (X = S and Y = S—compounds 1F). To investigate the role of the mutual interactions of the carbonyl groups, the fragments used in the search were expanded to include an extra carbonyl group (Figure 1d). The nature of the J atom in this group that precedes the ester/amide of Figure 1d defines the type of the derivative (ketone‐like, ester, or amide). The fragments identified were filtered to remove duplicates. The occurrence of n → π* interactions in these compounds was evaluated considering the geometric operational definition used by Raines & collaborators (Bartlett et al., 2010) (Figure 1a). In this framework, the interaction is present when Z…C = X angle (*α*) falls in the interval 99°–119° (along the Burgi–Dunitz trajectory (Burgi et al., 1973)), and the Z…C distance *d* ≤ 3.22 Å for Z = O and *d* ≤ 3.45 Å for Z = S (the sum of the van der Waals radii of oxygen/sulfur and carbon). Specifically, we searched for structure fragments where the distance d and the α angle lie within the range mentioned above. The number of fragments analyzed for each compound class is reported in Figure 1d and Table S1.

### 4.3 Analysis of protein data bank structures

The mutual influence of the n → π* interaction and the C pyramidalization was also explored in protein structures. To this aim, a nonredundant set (<25% pairwise sequence identity) of protein crystal structures with *R* < 20% and with a resolution of 1.0 Å or better was culled from the PDB of 20 November 2017 using the PISCES server (http://dunbrack.fccc.edu/PISCES.php) (Wang & Dunbrack Jr., 2003). We selected protein structures comprising more than 40 residues. The use of an eight‐year‐old release of the PDB was motivated by the observation that, in recent years, some refinement programs (Moriarty et al., 2014; Tronrud & Karplus, 2011) use as restraints backbone geometrical parameters that depend on the local conformation. The inclusion of recent structures in this study could have biased our analysis. On the other hand, the structures we used were refined using standard libraries of geometric parameters, which tend to somewhat restrict the amide group to planarity. Therefore, the trends we observe genuinely emerge, despite the protocols that tend to restrict the amide group to planarity, from the use of high‐resolution data in the refinements.

By excluding residues with multiple conformations, the search yielded 260 structures for a total of 36,534 backbone carbonyl carbon atoms whose pyramidalization, as defined in Section 4.1, was checked. The occurrence of the n → π* interaction was evaluated considering the preceding carbonyl carbon in the chain (at residue i‐1) as a possible lone‐pair donor. The interaction was assessed based on whether the geometrical restraints defined by Raines and collaborators (Figure 1a) (see also above) were satisfied. Secondary structure elements were assigned using the Kabsch and Sander method (Kabsch & Sander, 1983).

## AUTHOR CONTRIBUTIONS

**Luigi Vitagliano** and **Luciana Esposito:** Conceptualization; investigation; writing – original draft; writing – review and editing; methodology; formal analysis; data curation.

## CONFLICT OF INTEREST STATEMENT

The authors declare no conflicts of interest.

## Supporting information


**TABLE S1:** Details of the CSD search results for all the compounds in Figure 1d.
**TABLE S2:** Population of 10° ψ‐like bins for 2A/2B/3A/3B compounds. The populations are reported separately for both the “NO n → π*” and “n → π*” groups.
**TABLE S3:**. *p*‐Values from a two‐sample *t*‐test for the comparison of the *θ*_C_ means, calculated in each ψ‐like bin, for groups showing n → π* interactions versus groups not showing them (2A/2B/3A/3B compounds).
**TABLE S4:** α/β peptide structures (reported in Choudhary & Raines, 2011b) analyzed in Section 2.3.1 of the main text.
**TABLE S5:** Populations of the ψ bins plotted in Figure 6b.
**TABLE S6:** Populations of the ψ bins plotted in Figure 6c.
**FIGURE S1:** Carbon pyramidalization *θ*_C_ angle versus ψ‐like (ψ) dihedral angle in 3A compounds.
**FIGURES2:** Carbon pyramidalization *θ*_C_ angle versus ψ‐like (ψ) dihedral angle in 3B compounds.
**FIGURE S3:** Carbon pyramidalization *θ*_C_ angle versus ψ‐like (ψ) dihedral angle in 4A/4B compounds.
**FIGURE S4:** Cooperative effects of consecutive carbonyl groups on carbon pyramidalization.

## Data Availability

The data that supports the findings of this study are available in the supplementary material of this article.
