# Supplementary material for: The interplay between the pyramidalization of carbonyl carbons and the n → π* interactions in biomolecules
Source: Protein Sci. 2026 May 4;35(6):e70602. doi: 10.1002/pro.70602 (PMC13137273; doi:10.1002/pro.70602)
Supplement: Supplementary file 1 — TABLE S1: Details of the CSD search results for all the compounds in Figure 1d. TABLE S2: Population of 10° ψ‐like bins for 2A/2B/3A/3B compounds. The populations are reported separately for both the “NO n → π*” and “n → π*” groups. TABLE S3:. p‐Values from a two‐sample t‐test for the comparison of the θ C means, calculated in each ψ‐like bin, for groups showing n → π* interactions versus groups not showing them (2A/2B/3A/3B compounds). TABLE S4: α/β peptide structures (reported in Choudhary & Raines, 2011b) analyzed in Section 2.3.1 of the main text. TABLE S5: Populations of the ψ bins plotted in Figure 6b. TABLE S6: Populations of the ψ bins plotted in Figure 6c. FIGURE S1: Carbon pyramidalization θ C angle versus ψ‐like (ψ) dihedral angle in 3A compounds. FIGURES2: Carbon pyramidalization θ C angle versus ψ‐like (ψ) dihedral angle in 3B compounds. FIGURE S3: Carbon pyramidalization θ C angle versus ψ‐like (ψ) dihedral angle in 4A/4B compounds. FIGURE S4: Cooperative effects of consecutive carbonyl groups on carbon pyramidalization. [file PRO-35-e70602-s001.docx]

**Supplementary Information**

**The Interplay Between the Pyramidalization of Carbonyl Carbons and the n→π* Interactions in Biomolecules**

Luigi Vitagliano^*^ and Luciana Esposito^*^

Institute of Biostructures and Bioimaging, CNR, 80131 Naples, Italy.

^*^Corresponding authors

E-mails: luigi.vitagliano@cnr.it, luciana.esposito@cnr.it

**Table S1. Details of the CSD search results for all the compounds in Figure 1D.**

| **Compound** | **Z** | **J** | **X** | **Y** | **#Fragments (Entries)** | **#n**→**π*/NO n**→**π*** | **<ABSθ> n**→**π*/NO n**→**π*** |
| --- | --- | --- | --- | --- | --- | --- | --- |
| 2A | **O** | NH | **O** | O | 1478 (1144) | 316/1162 | 3.57/1.72 |
| 2B | **O** | NH | **O** | N | 2727 (1339) | 900/1827 | 3.24/2.33 |
| 2C | **O** | NH | **O** | S | 4 (3) |  |  |
| 3A | **O** | C | **O** | O | 2988 (2039) | 371/2617 | 2.65/1.79 |
| 3B | **O** | C | **O** | N | 2403 (1337) | 154/2249 | 2.34/1.47 |
| 3C | **O** | C | **O** | S | 7 (6) |  |  |
| 4A | **O** | O | **O** | O | 539 (437) | 39/500 | 2.90/1.94 |
| 4B | **O** | O | **O** | N | 333 (248) | 47/286 | 3.03/1.96 |
| 4C | **O** | O | **O** | S | 1 (1) |  |  |
| 5A | **O** | NH | **S** | O | 0 |  |  |
| 5B | **O** | NH | **S** | N | 31 (25) | 14/17 | 4.29/2.03 |
| 5C | **O** | NH | **S** | S | 6 (5) |  |  |
| 6A | **O** | C | **S** | O | 1 (1) |  |  |
| 6B | **O** | C | **S** | N | 2 (1) |  |  |
| 6C | **O** | C | **S** | S | 0 |  |  |
| 7A | **O** | O | **S** | O | 0 |  |  |
| 7B | **O** | O | **S** | N | 0 |  |  |
| 7C | **O** | O | **S** | S | 0 |  |  |
| 8A | **S** | NH | **O** | O | 36 (31) | 6/30 | 5.43/1.80 |
| 8B | **S** | NH | **O** | N | 69 (62) | 7/62 | 6.40/1.94 |
| 8C | **S** | NH | **O** | S | 0 |  |  |
| 9A | **S** | C | **O** | O | 11 (10) |  |  |
| 9B | **S** | C | **O** | N | 2 (1) |  |  |
| 9C | **S** | C | **O** | S | 0 |  |  |
| 10A | **S** | O | **O** | O | 6 (6) |  |  |
| 10B | **S** | O | **O** | N | 2 (2) |  |  |
| 10C | **S** | O | **O** | S | 0 |  |  |
| 11A | **S** | NH | **S** | O | 0 |  |  |
| 11B | **S** | NH | **S** | N | 3 (3) |  |  |
| 11C | **S** | NH | **S** | S | 0 |  |  |
| 12A | **S** | C | **S** | O | 0 |  |  |
| 12B | **S** | C | **S** | N | 5 (3) |  |  |
| 12C | **S** | C | **S** | S | 0 |  |  |
| 13A | **S** | O | **S** | O | 0 |  |  |
| 13B | **S** | O | **S** | N | 0 |  |  |
| 13C | **S** | O | **S** | S | 0 |  |  |

**Table S2. Population of 10° ψ-like bins for 2A/2B/3A/3B compounds. The populations are separately reported for both “NO n→π*” and “n→π*” groups.**

|  | **Population** | | | | | | | |
| --- | --- | --- | --- | --- | --- | --- | --- | --- |
| **ψ-like bin (°)** | **2A** | | **2B** | | **3A** | | **3B** | |
|  | **NO n**→**π*** | **n**→**π*** | **NO n**→**π*** | **n**→**π*** | **NO n**→**π*** | **n**→**π*** | **NO n**→**π*** | **n**→**π*** |
| -170 | **111** | 0 | **58** | 0 | **207** | 0 | **29** | 0 |
| -160 | **55** | 0 | **39** | 0 | **148** | 0 | **24** | 0 |
| -150 | **31** | 3 | **42** | 4 | **146** | 8 | **33** | 2 |
| -140 | **18** | 9 | **39** | **10** | **83** | **17** | **45** | **14** |
| -130 | 6 | 3 | **39** | **53** | **68** | **11** | **71** | **37** |
| -120 | 4 | 2 | **24** | **10** | **54** | 8 | **68** | **32** |
| -110 | 2 | 0 | **12** | 0 | **33** | 5 | **41** | 2 |
| -100 | 1 | 0 | **11** | 0 | **31** | 4 | 8 | 0 |
| -90 | 5 | 0 | 7 | 0 | **41** | 6 | **16** | 1 |
| -80 | 4 | 0 | **11** | 0 | **66** | 3 | **20** | 0 |
| -70 | 2 | 0 | **14** | 0 | **67** | 5 | **11** | 0 |
| -60 | **16** | 0 | **34** | 0 | **75** | **27** | 8 | 3 |
| -50 | **20** | **50** | **55** | **53** | **57** | **31** | 4 | 0 |
| -40 | **22** | **46** | **27** | **197** | **41** | **41** | 6 | 0 |
| -30 | **13** | **34** | **73** | **175** | **31** | **10** | 6 | 2 |
| -20 | **32** | 4 | **134** | **25** | **29** | 3 | **72** | 4 |
| -10 | **34** | 0 | **114** | 0 | **28** | 0 | **276** | 0 |
| 0 | **21** | 0 | **96** | 0 | **20** | 0 | **742** | 0 |
| 10 | **29** | 0 | **73** | 0 | **20** | 0 | **250** | 0 |
| 20 | **24** | 1 | **81** | 6 | **29** | 2 | **67** | 1 |
| 30 | **14** | **14** | **50** | **103** | **34** | **18** | 8 | 0 |
| 40 | **21** | **54** | **17** | **103** | **41** | **48** | 4 | 0 |
| 50 | **19** | **42** | **30** | **38** | **70** | **35** | 0 | 0 |
| 60 | 9 | 5 | **28** | 4 | **79** | **21** | 7 | 0 |
| 70 | 8 | 0 | **15** | 0 | **79** | **18** | **10** | 0 |
| 80 | 9 | 0 | **16** | 0 | **60** | 5 | **16** | 2 |
| 90 | 0 | 0 | **19** | 0 | **39** | 5 | **16** | 2 |
| 100 | 2 | 0 | **34** | 0 | **36** | 3 | **15** | 1 |
| 110 | 2 | 0 | **42** | 0 | **39** | 3 | **37** | 2 |
| 120 | **10** | 0 | **69** | **12** | **43** | 5 | **77** | **11** |
| 130 | **19** | 7 | **75** | **58** | **49** | **10** | **80** | **28** |
| 140 | **33** | **22** | **72** | **35** | **71** | **13** | **31** | 9 |
| 150 | **76** | **16** | **123** | **14** | **150** | 6 | **42** | 1 |
| 160 | **129** | 4 | **120** | 0 | **148** | 0 | **25** | 0 |
| 170 | **183** | 0 | **64** | 0 | **199** | 0 | **34** | 0 |
| 180 | **176** | 0 | **59** | 0 | **203** | 0 | **49** | 0 |

Bin populations with less than 10 measures are coloured gray and not considered in plots of Figure 3C,4C.

**Table S3. P-values from a two-sample t-test for the comparison of the θ_C_ means, calculated in each ψ-like bin, for groups showing n→π* interactions vs. groups not showing them (2A/2B/3A/3B compounds).**

| **Compound** | **ψ-like bin (°)** | | | | | | | | | | | | | | | | |
| --- | --- | --- | --- | --- | --- | --- | --- | --- | --- | --- | --- | --- | --- | --- | --- | --- | --- |
|  | **-140** | **-130** | **-120** | **-60** | **-50** | **-40** | **-30** | **-20** | **30** | **40** | **50** | **60** | **70** | **120** | **130** | **140** | **150** |
| **2A** |  |  |  |  | 4.4*  10^-7^ | 3.8*  10^-4^ | NO |  | NO | 3.5*  10^-7^ | 2.7*  10^-9^ |  |  |  |  | NO | NO |
| **2B** | NO | 1.1*  10^-6^ | 6.1*  10^-3^ |  | 1.7*  10^-5^ | 1.8*  10^-5^ | NO | 5.4*  10^-7^ | NO | 3.2*  10^-6^ | 4.7*  10^-7^ |  |  | 5.4*  10^-3^ | NO | NO | NO |
| **3A** | NO | NO |  | 6.6*  10^-8^ | 7.4*  10^-5^ | 7.6*  10^-4^ | NO |  | NO | 5.2*  10^-3^ | 1.5*  10^-9^ | 1.9*  10^-11^ | 7.7*  10^-5^ |  |  | NO | NO |
| **3B** | NO | 4.1*  10^-3^ | 9.0*  10^-3^ |  |  |  |  |  |  |  |  |  |  | NO | NO |  |  |

The statistical significance of the mean difference is evaluated by a two-sample t-test at a significance level of 0.05. The test has been performed for bins where the population of the two groups (NO n→ π* and n→ π*) is at least 10 (see Tabel S2 for the population data per bin). The label “NO” is reported when the mean differences are not statistically significant.

**Table S4.** **α/β peptide structures reported in Choudhary and Raines, Protein Sci. 2011b and analysed in section 2.3.1 of the main text.**

| **Cambridge Crystallographic Data Centre refcodes** |
| --- |
| OGATAS, CAXRID, OGASOF, OGASUL, OGATEW, OGATIA, COVFUP, OGATOG, OGATUM, OGAVAU, OGAVEY, OGAVIC, OGAVOI, COVGAW, PUCCIA, PUCCOG, PUCCUM, PUCDEX, PUCDUN |

**Table S5. Populations of the ψ bins plotted in Figure 6B.**

|  | **Population** | |
| --- | --- | --- |
| **ψ bin (°)** | **NO n**→**π*** | **n**→**π*** |
| -170 | **134** | 0 |
| -160 | **44** | 0 |
| -150 | 13 | 0 |
| -140 | 15 | 0 |
| -130 | 10 | 12 |
| -120 | 4 | 2 |
| -110 | 7 | 4 |
| -100 | 13 | 0 |
| -90 | 11 | 0 |
| -80 | 16 | 2 |
| -70 | 26 | 2 |
| -60 | **166** | 3 |
| -50 | **1814** | **1161** |
| -40 | **450** | **5781** |
| -30 | **369** | **2024** |
| -20 | **1339** | **430** |
| -10 | **1457** | 1 |
| 0 | **1052** | 0 |
| 10 | **773** | 3 |
| 20 | **473** | 4 |
| 30 | **180** | **169** |
| 40 | **86** | **255** |
| 50 | **116** | **120** |
| 60 | **199** | 4 |
| 70 | **210** | 2 |
| 80 | **214** | 1 |
| 90 | **245** | 0 |
| 100 | **451** | 0 |
| 110 | **968** | 3 |
| 120 | **1942** | **147** |
| 130 | **2524** | **885** |
| 140 | **2543** | **852** |
| 150 | **2586** | **279** |
| 160 | **2404** | 17 |
| 170 | **1133** | 2 |
| 180 | **380** | 2 |

Bin populations with less than 40 measures are coloured gray and not considered in plot of Figure 6B.

**Table S6. Populations of the ψ bins plotted in Figure 6C.**

|  | **Population** | |
| --- | --- | --- |
| **ψ bin (°)** | **NO n**→**π***  **& NO SS** | **n**→**π***  **& NO SS** |
| -60 | **7** | 0 |
| -50 | **21** | **5** |
| -40 | **21** | **30** |
| -30 | **40** | **37** |
| -20 | **110** | **9** |
| -10 | **166** | **0** |
| 0 | **155** | **0** |


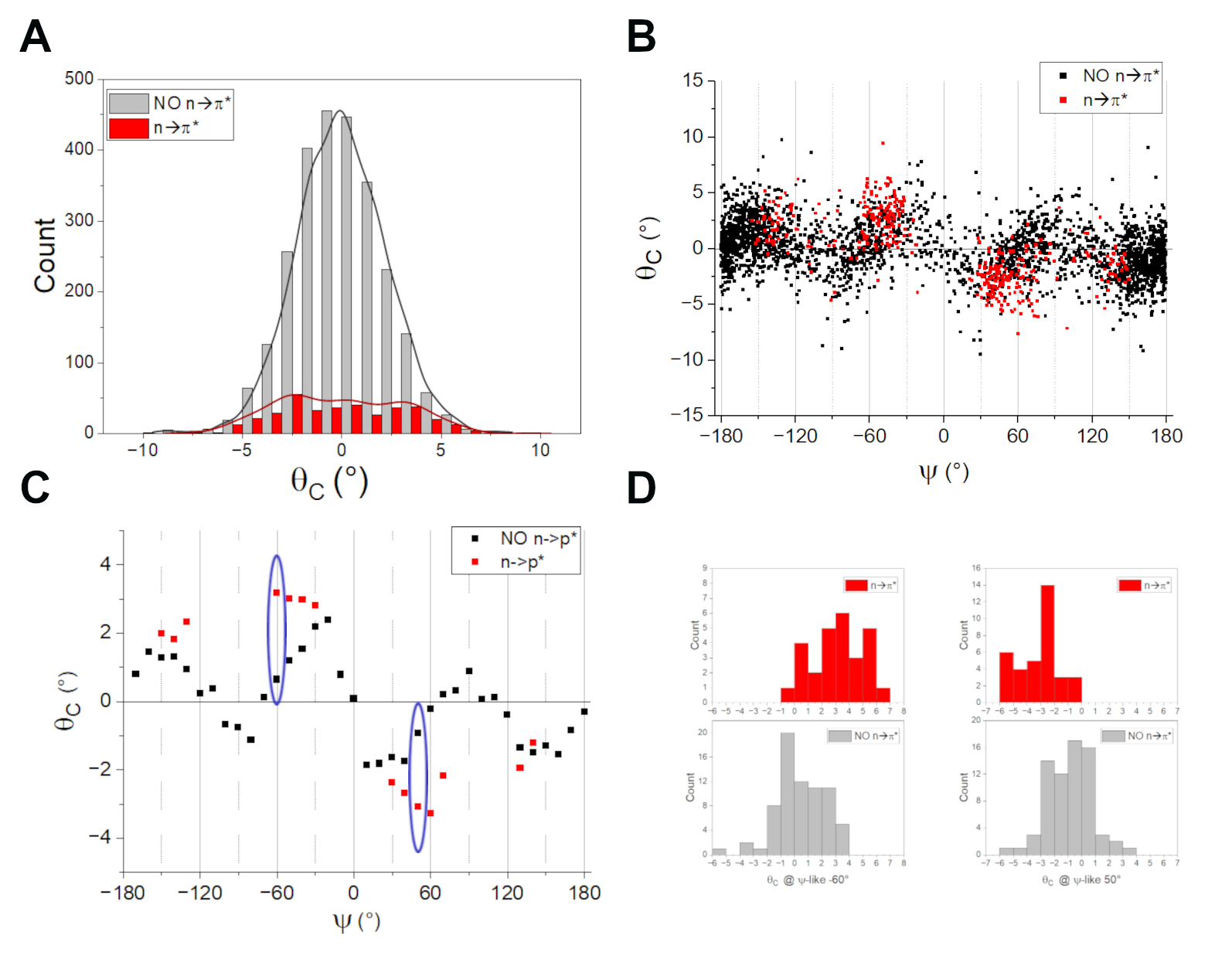


**Figure S1**

Carbon pyramidalization θ_C_ angle vs. ψ-like (ψ) dihedral angle in 3A compounds. (A) Distributions of θ_C_ values for the two classes of carbonyls (in red are the carbonyls which have a nearby carbonyl group establishing n**→**π* interactions, in black those which have not, NO n**→**π*). A fitting curve (kernel smoothing) is superimposed to the histograms; (B) Plot of θ_C_ vs. ψ-like for all the fragments selected; (C) averaged pyramidalization per bin (10°) for points in panel B (only bins containing more than 10 measures are considered). Selected different values for the two classes in the same bin are circled in blue and analysed in panel D; (D) θ_C_ distributions for the ψ= -60° and ψ=50° bins of panel C for both carbonyls displaying n**→**π* interactions (red-upper panel) and carbonyls not displaying them (gray-lower panel). See Tables S1-S3 for details on the populations of the overall datasets (Table S1), of the plotted bins (Table S2), as well as on the statistical significance of the mean differences (Table S3).


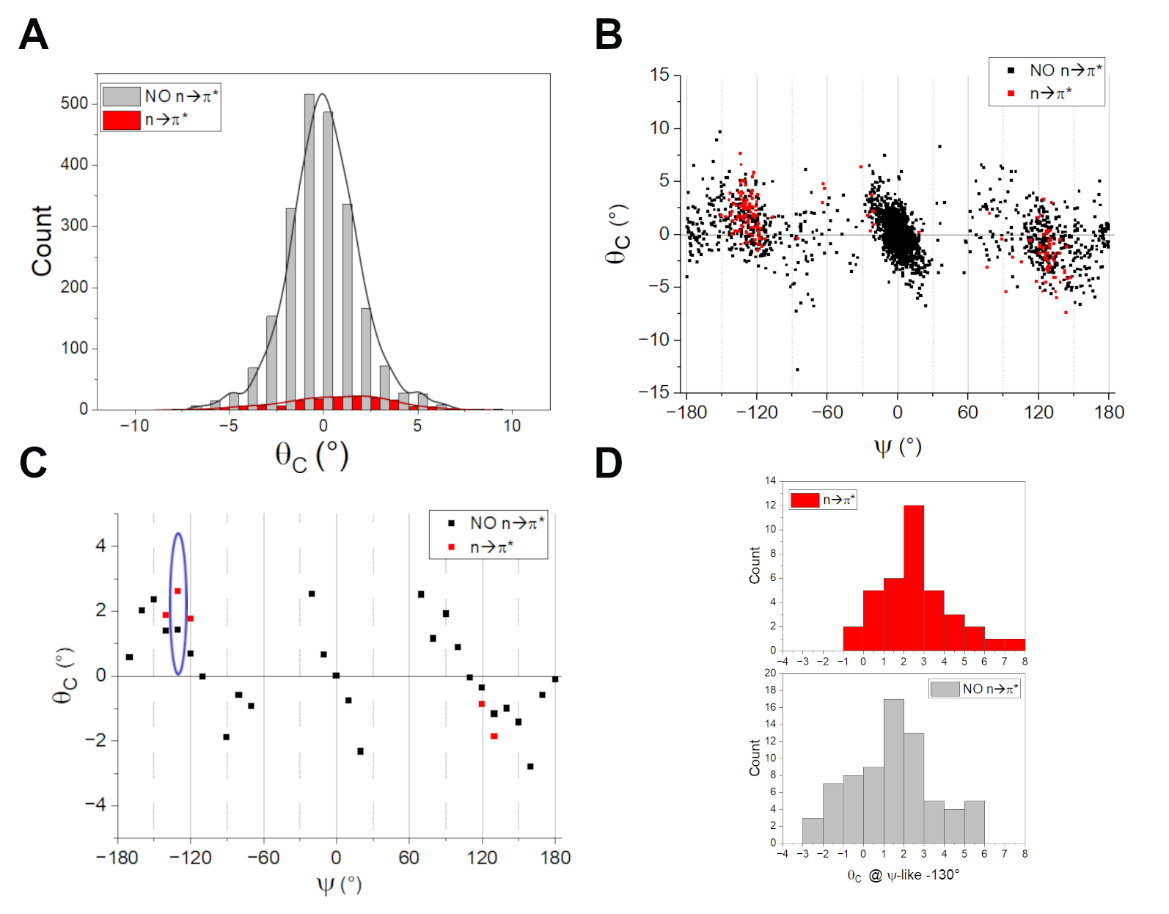


**Figure S2**

Carbon pyramidalization θ_C_ angle vs. ψ-like (ψ) dihedral angle in 3B compounds. (A) Distributions of θ_C_ values for the two classes of carbonyls (in red are the carbonyls which have a nearby carbonyl group establishing n**→**π* interactions, in black those which have not, NO n**→**π*). A fitting curve (kernel smoothing) is superimposed to the histograms; (B) Plot of θ_C_ vs. ψ-like for all the fragments selected; (C) averaged pyramidalization per bin (10°) for points in panel B (only bins containing more than 10 measures are considered). Selected different values for the two classes in the same bin are circled in blue and analysed in panel D; (D) θ_C_ distributions for the ψ= -130° bin of panel C for both carbonyls displaying n**→**π* interactions (red-upper panel) and carbonyls not displaying them (gray-lower panel). See Tables S1-S3 for details on the populations of the overall datasets (Table S1), of the plotted bins (Table S2), as well as on the statistical significance of the mean differences (Table S3).


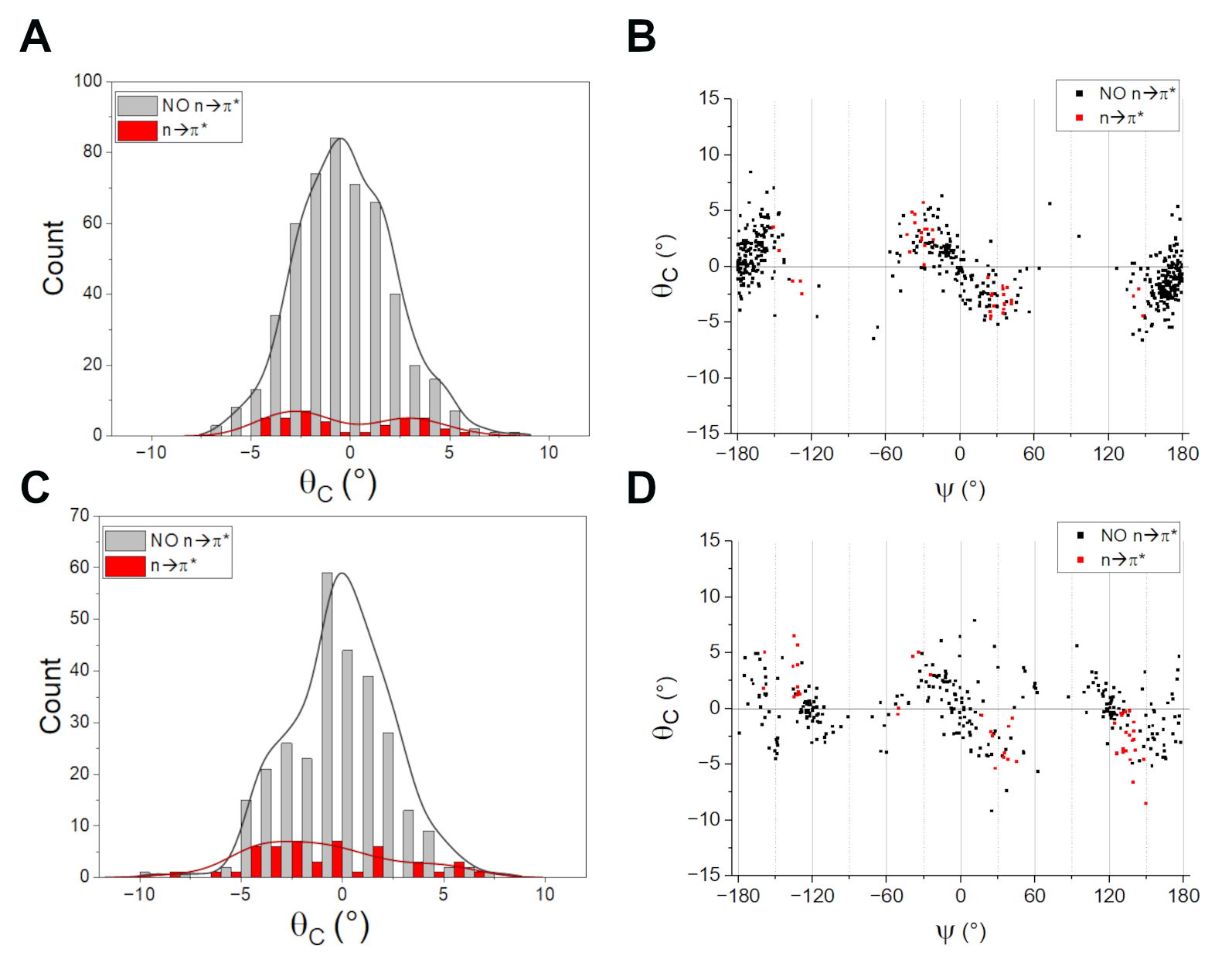


**Figure S3**

Carbon pyramidalization θ_C_ angle vs. ψ-like (ψ) dihedral angle in 4A/4B compounds. (A) Compound 4A distributions of θ_C_ values for the two classes of carbonyls (in red are the carbonyls which have a nearby carbonyl group establishing n**→**π* interactions, in black those which have not, NO n**→**π*). A fitting curve (kernel smoothing) is superimposed to the histograms; (B) Compound 4A plot of θ_C_ vs. ψ-like for all the fragments selected; (C) Compound 4B distributions of θ_C_ values for the two classes of carbonyls (in red are the carbonyls which have a nearby carbonyl group establishing n**→**π* interactions, in black those which have not, NO n**→**π*). A fitting curve (kernel smoothing) is superimposed on the histograms; (B) Compound 4B plot of θ_C_ vs. ψ-like for all the fragments selected; See Tables S1 for details on the populations of the overall datasets.


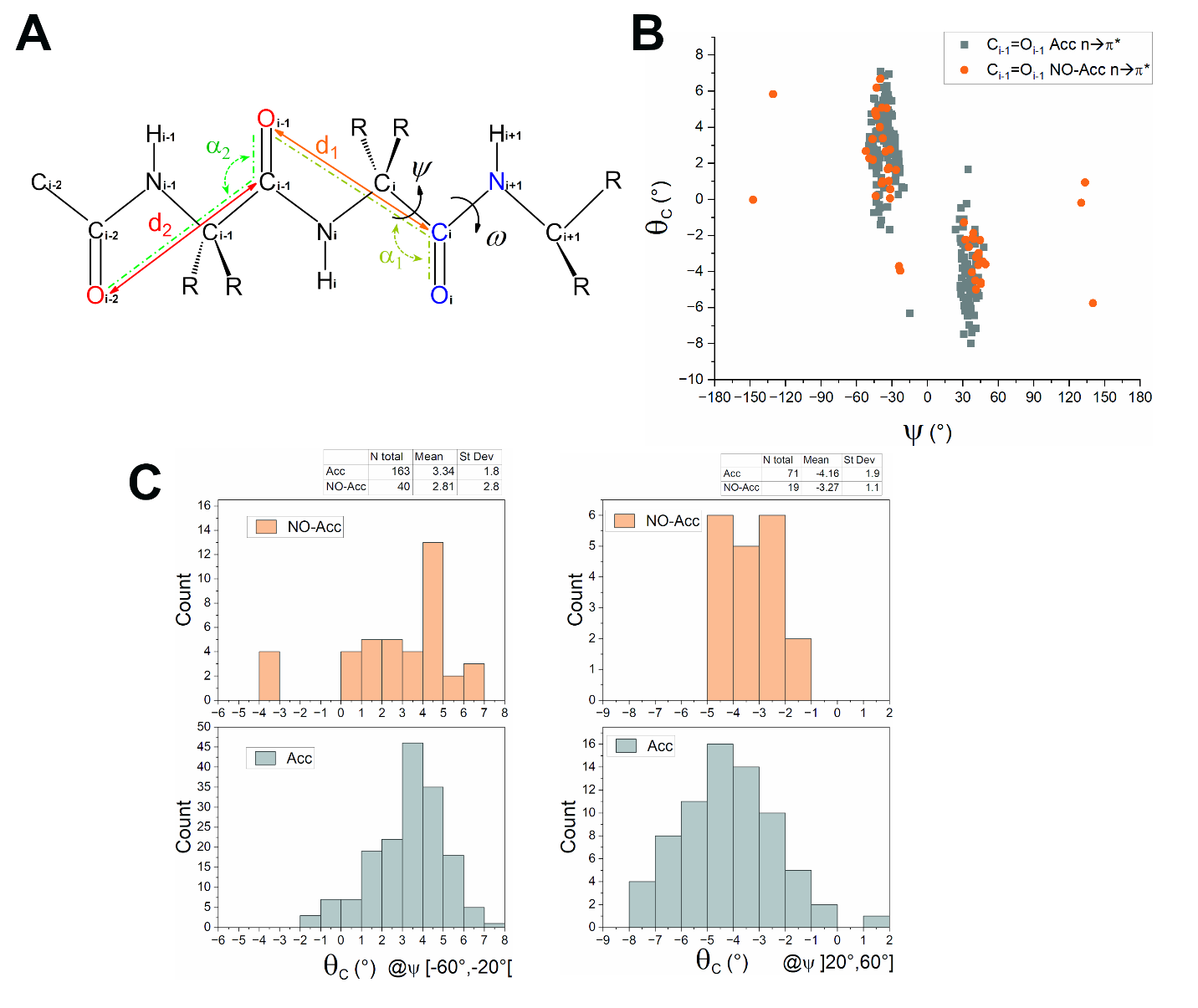


**Figure S4**

Cooperative effects of consecutive carbonyl groups on carbon pyramidalization. (A) Fragments searched for in the CSD following the same filters used in previous searches (see Materials and Methods section 4.2 of the main text). We analysed the pyramidalization of C_i_ carbonyl carbon atoms (in blu) under the condition that the preceding carbonyl C_i-1_=O_i-1_ is a n→π* donor (i.e. d_1_ ≤ 3.22 Å and 99°≤ α_1_ ≤ 119°); (B) θ_C_ pyramidalization of C_i_ atoms vs. ψ angle. We separately analysed fragments where the carbonyl C_i-1_=O_i-1_ n→π* donor is either acceptor of n→π* interaction from C_i-2_=O_i-2_ (i.e. d_2_ ≤ 3.22 Å and 99°≤ α_2_ ≤ 119°) (grey squares) or not (orange circle); (C) Distributions of θ_C_ angles in two populated regions of ψ for the two groups of measures (see panel B). Some statistical data for the two sets of measures are reported in the tables at the top of the distribution plots.
